# Case of Unusual Foreign Body in the Rectum

**DOI:** 10.4103/1319-3767.48973

**Published:** 2009-04

**Authors:** Murtaza A. Akhtar, Pooja K. Arora

**Affiliations:** Department of Surgery, NKP Salve Institute of Medical Sciences and Research, Digdoh Hills, Hingna Road, Nagpur - 440 026, Maharashtra, India

**Keywords:** Foreign body, rectal trauma, sexual perversions

## Abstract

A 44-year-old male patient with a foreign body in rectum (beverage bottle), introduced as sexual perversion, is presented with literature review. The management emphasis is on transanal retrieval and ruling out of the rectal and colonic perforation and the requirement for postremoval psychiatric treatment.

Foreign body within the rectum occurs infrequently. Majority of objects are introduced through anus; however, sometimes a foreign body is swallowed, passes through the gastrointestinal tract and is held up in rectum. They are known for potential complications and present as a challenge to clinical management. They should be seriously and expeditiously treated.

## CASE REPORT

**Figure 1:**
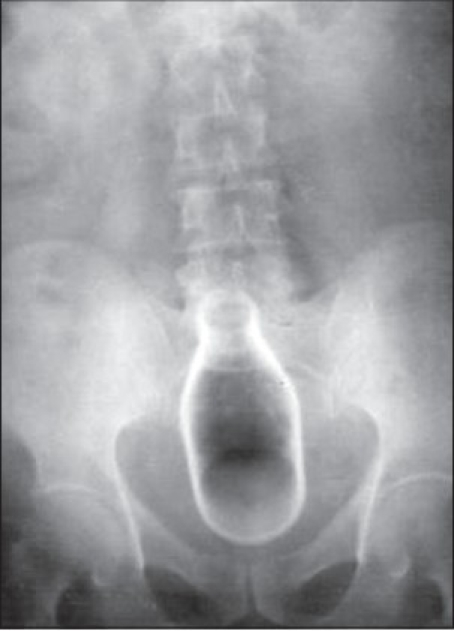
X-ray abdomen showing foreign body

A 44-year-old male presented with the history of introducing a beverage bottle in the rectum and bleeding per rectum since one day. The failure of repeated attempts of self-removal brought the patient to the hospital. He gave history of similar attempts of using objects for sexual gratification in past. Vital signs were normal. Abdomen was soft. Foreign body was not palpable per abdomen. X-ray pelvis showed the bottle in lower abdomen and pelvis [Figure 1]. Per rectal examination performed after the X-ray of the abdomen, revealed the base of the glass bottle.

The manual removal by holding the base of the bottle was impossible and snares repeatedly slipped due to mucous coating the surface. Moreover, the bottle could not be manipulated upside down in the rectum due to its large size. After exhausting all methods described in the literature, a novel way of bearing the bottle down was attempted and was successful. After reassurance and IV analgesic, in lithotomy position, patient was encouraged to bear down as if he is pushing the faeces. As the bottom of bottle showed up at anal verge, it was grasped by an obstetrics forceps and was removed with gentle traction. Postremoval per rectal examination and sigmoidoscopy did not reveal any colorectal injury except some minor anal tears. As patient was a habitual pervert, no major anal tears were noticed. Postremoval recovery was uneventful and patient did not have anal incontinence or perianal infection. He was referred for psychiatric treatment.

## DISCUSSION

Reports of foreign body within the rectum are uncommon in Asia, and the majority of case series are reported from Eastern Europe. [1–6] Males are commonly affected.[1,2] The age group is 16-80 years;[1] however, there is a bimodal age distribution, observed in the twenties for anal erotism or forced introduction through anus, and in the sixties mainly for prostatic massage and breaking fecal impactions. The foreign bodies commonly reported were plastic or glass bottles, cucumbers, carrots, wooden, or rubber objects.[2] Other objects reported are bulb, tube light, axe handle, broomstick, vibrators, etc. The object length varied between 6 and 15 cm, and larger objects were more prone for complications.[2]

Abdominal and rectal pains, bleeding per rectum are the common presenting symptoms. Per rectal examination is the cornerstone in the diagnosis, but it should be performed after X-ray abdomen to prevent accidental injury to the surgeon from sharp objects. X-ray pelvis and X-ray abdomen help in locating and localizing the foreign body and also rule out intestinal perforation. The lateral films of pelvis will orient whether the foreign body is high or low lying.

Majority (90%) of the cases is treated by transanal retrieval.[1,2,6,7] Hard objects are potentially traumatic and tend to migrate upwards.[8] Abdominal manipulation and stabilization helps in retrieval when the bottle is slippery. Obstetric forceps or snares are only helpful in grasping the broad and slippery base with limited success. Colonoscopy removal is also reported with good success.[3] However, limited studies in the literature restrict the definition of the major role of colonoscopy. Laparotomy is only required in impacted foreign body and or with perforation peritonitis. Even with laparotomy, the aim is transanal removal and closure of perforation with diversion colostomy. Postretrieval colonoscopy is mandatory to rule out colorectal injury.

In the present case, transanal removal was carried out and the only difficulty was grasping of bottle base with fingers and snares due to mucous and slippery base. Asking the patient to push down the foreign body and grasping the base of bottle with obstetric forceps, which gave a firm grip over the base, helped us to overcome this difficulty. Patient was referred to the psychiatrist for his perversion disorder, which was also mandatory for preventing recurrences.
